# An interdisciplinary student-led multifaceted intervention addressing overuse of broad-spectrum antibiotics for patients with penicillin allergies

**DOI:** 10.1186/s13756-023-01232-0

**Published:** 2023-04-15

**Authors:** Bryana Banashefski, Philip Henson, Navindra David, Hui Ting Kok, Frans J. Beerkens, Margaret Shyu, Anne S. Linker, Surafel Tsega, Andrew Dunn, Risa Fuller

**Affiliations:** 1 Icahn School of Medicine at Mount Sinai, 1468 Madison Ave, New York, NY 10029 USA; 2 Phillips School of Nursing at Mount Sinai Beth Israel, New York, NY USA; 3 Division of Hospital Medicine, Department of Medicine at Mount Sinai, New York, NY USA; 4 Division of Infectious Diseases, Department of Medicine, Icahn School of Medicine at Mount Sinai, New York, NY USA

**Keywords:** Quality intervention, β-lactams, Penicillin allergy

## Abstract

**Background:**

Though 15% of hospitalized patients have a documented penicillin (PCN) allergy, fewer than 1% have an IgE-mediated reaction that necessitates avoidance of β-lactam antibiotics.

**Objective:**

Our interdisciplinary team of medical and nursing students led and executed a two-pronged quality improvement intervention to reduce prescribing of non-β-lactam antibiotics (NBLs) for patients with reported PCN allergies. To the best of our knowledge, this is the first multidisciplinary student-led intervention aimed at educating providers on low-risk penicillin allergy and encouraging best antibiotic prescribing practices.

**Design and participants:**

The intervention took place from June 2021 to February 2022. We developed and provided clinician education modules, including peer-to-peer information sharing and in-person small group discussions, as well as clinical decision support (CDS) strategies through the electronic medical record (EMR). The target population was attendings, residents, nurse practitioners, and physician assistants on the hospital medicine service at a large urban academic tertiary care center. We followed the SQUIRE 2.0 guidelines for reporting on quality improvement.

**Main measures:**

Primary outcome measures included number of NBL prescriptions and use of nonspecific descriptors (e.g., “other” or “unknown”) for PCN allergy reaction type, and were compared with a pre-intervention period.

**Key results:**

The percent of β-lactam prescriptions for patients with a PCN allergy after the intervention increased from 19 to 23% (*p* = 0.006). For patients with a low severity PCN allergy, the percent of β-lactam prescriptions increased from 20 to 28% (*p* = 0.001). There was a significant decrease in nonspecific PCN allergy reaction type from 23% in the pre-intervention period to 20% post-intervention (*p* = 0.012).

**Conclusions:**

An intervention focused on educating prescribers and CDS strategies delivered through the EMR increased appropriate β-lactam prescribing for patients with a documented low-risk PCN allergy and reduced the use of nonspecific PCN allergy reaction type in EMR documentation.

**Supplementary Information:**

The online version contains supplementary material available at 10.1186/s13756-023-01232-0.

## Introduction

Penicillin and its derivatives are widely used worldwide [1–3]. Use of penicillins is associated with adverse IgE-mediated hypersensitivity reactions, such as hives, pruritus, or anaphylaxis [4]. Incomplete or inaccurate documentation of such allergies in the medical record can impact physician prescribing behavior. Providers are often reluctant to order β-lactam antibiotics when evaluating patients when documentation of penicillin allergy lacks detail about the specific reaction symptoms [5]. While 10% of hospitalized patients have a documented penicillin allergy, only 1% of listed allergies are IgE-mediated reactions that require avoidance of penicillin [6].

Inaccurate documentation of penicillin allergies negatively impacts patients and the healthcare system [7]. Individuals with penicillin allergies have 23% more *Clostridioides difficile* infections, 14% more methicillin-resistant *Staphylococcus aureus* (MRSA) infections, and 30% more vancomycin-resistant enterococcus (VRE) infections than matched controls [8]. Physicians tend to use alternative antibiotics such as fluoroquinolones, carbapenems, and vancomycin in order to avoid potential allergic reactions, although such medications may be less effective against the target organism and associated with more toxicity [6, 9]. From a public health standpoint, inappropriate use of broad-spectrum antibiotics increases the prevalence of drug-resistant bacteria. In addition, use of non-β-lactam antibiotics (NBLs) increases inpatient medication cost by an average of $600 per person per infection and by an average of $200 in outpatient medication cost per person per infection [10].

In light of the growing awareness of the consequences of incorrect penicillin allergy documentation, health care organizations have attempted to create processes to remove inappropriate allergy designations [11, 12]. Educational interventions have focused on educating providers on accurate assessment and documentation of the patient’s allergy history [13], overuse of antibiotics, and appropriate prescribing practices [14]. However, education-only interventions on antimicrobial stewardship have rarely resulted in sustained change [15]. Prior work suggests that electronic medical record (EMR) interventions, including improved data tracking and development of best practice advisories, may augment educational interventions to sustain the impact of improved allergy labeling efforts [15].

The primary purpose of our study was to reduce prescribing of unnecessary NBLs for patients with listed penicillin allergies through clinician education and clinical decision support (CDS) strategies through the EMR. The educational and CDS interventions were developed and implemented with the goal of encouraging provider reassessment of listed penicillin allergy severity and enhancing awareness of appropriate antibiotic prescribing practices for patients with a documented penicillin allergy. To the best of our knowledge this is the first multidisciplinary, student-led intervention aimed at educating providers on appropriate documentation of low-risk penicillin allergies and encouraging best practice in antibiotic prescribing.

## Methods

### Intervention

Our intervention targeted prescribers (attendings, residents, nurse practitioners, and physician assistants) on the hospital medicine service at an 1,100-bed urban academic tertiary care center. The intervention took place from June 7, 2021 to February 25, 2022. The pre-intervention period was defined as July 2019 to May 2021 in order to capture data prior to the COVID-19 pandemic. June 2021 marked the beginning of the intervention for both the educational and CDS components. The educational intervention was live from June to July 2021. One component of the CDS intervention (the Best Practice Advisory (BPA) was live from June 2021-October 2021; the second component of the CDS intervention (embedded link to prescribing best practices) was live from June 2021-present. Data collection ended in February 2022. The study was planned and executed as part of the Student High Value Care Curriculum at the Icahn School of Medicine at Mount Sinai. The project was led by a group of medical and nursing students, with mentors in Infectious Disease and Hospital Medicine.

The educational component (June and July 2021) included virtual didactics paired with weekly in-person marketing campaigns for resident physicians, nurse practitioners, and physician assistants. The virtual didactic sessions consisted of 10-min presentations to attendings, residents, nurses, and physician assistants during Hospital Medicine Grand Rounds and resident noon conferences. The virtual presentation included a description of the high prevalence of documented penicillin allergies compared to the prevalence of IgE-mediated penicillin allergies in the population, as well as an explanation of the adverse effects patients can experience when NBLs are prescribed unnecessarily. The presentation also included a description of the best practices for antibiotic prescribing, represented as an algorithm developed by the antibiotic stewardship committee (included in an Additional file 1) and an overview of the intervention’s EMR changes (described below) and how these changes can aid in decision-making for antibiotic prescribing. The in-person marketing campaign included weekly one-hour sessions during which a project team member hosted conversations in the resident, physician assistant, and nurse practitioner lounges about the project and the importance of antibiotic stewardship for patients with a documented penicillin allergy. The project team member provided food and coffee as well as handouts that highlighted key takeaways for the project. In addition, two resident physicians helped conduct peer-to-peer communication to enhance awareness.

**Fig. 1:**
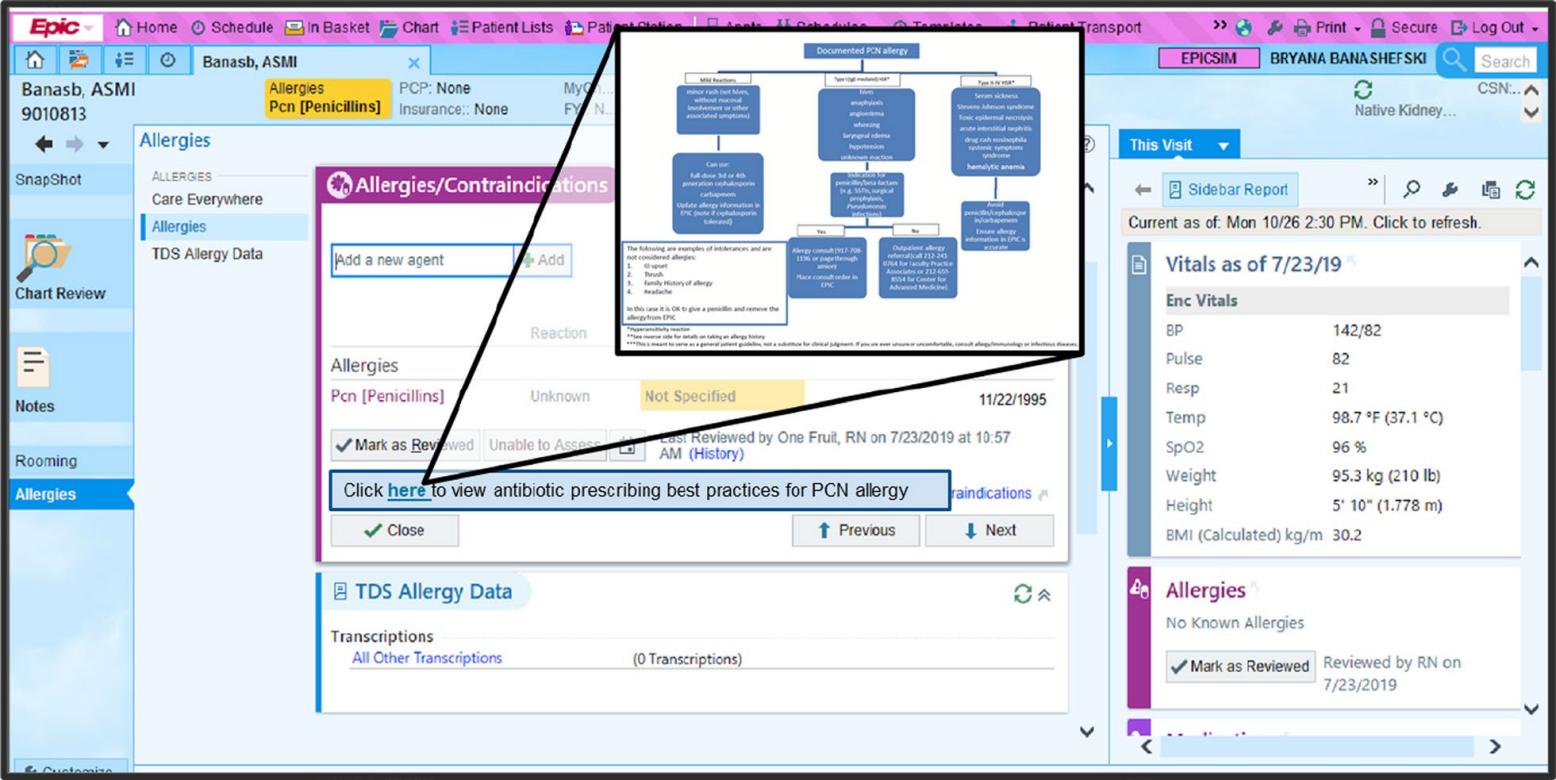
Allergy history page in Epic that shows the new hyperlink that embeds a decision tree for best practices when choosing an antibiotic for patients with a penicillin allergy. This link only appears in the allergy history for patients with a penicillin allergy. The callout in Fig. 1 is included an Additional file 1

The CDS component (June 2021–February 2022) included two interventions. The first CDS intervention (June 2021–February 2022) included a new link to antibiotic prescribing best practices embedded in the allergy section within the EMR (Fig. 1). The best practices document was handed out and reviewed during the educational sessions. Embedding the link in the allergy section of the EMR for patients with PCN allergies provided real-time access to best practice prescribing methods.

The second CDS intervention (June–October 2021) was a β-lactam history BPA created to notify providers ordering an NBL for a patient with a reported penicillin allergy. The BPA specified whether the patient had tolerated a β-lactam antibiotic in the past without a high severity reaction. If the patient met such criteria, the BPA would appear within the provider’s order workflow with a table that included the name, dose, and date the β-lactam was previously administered. The BPA was initially implemented as a pilot focusing on aztreonam since it is often used as a broad spectrum alternative for patients with penicillin allergies. The following BPA data were collected and analyzed: BPA alert description, alert date/time, user triggered by the alert, and alert-triggered orders. As a measure of safety, we monitored utilization of the anaphylaxis order set after the BPA fired. The anaphylaxis order set includes all medications necessary for treatment of anaphylaxis.

### Data acquisition

Data for analysis were obtained from Epic Systems software (Epic, Verona, WI). Baseline data were obtained from July 2019 to May 2021. Each entry in the data set represented an antibiotic dose administered to an admitted patient with a documented penicillin allergy. Variables captured included patient sex, age, hospital admission date/time, hospital unit, antibiotic order, antibiotic order date/time, ordering provider, penicillin allergy status, penicillin allergy reaction, penicillin allergy severity, the date the penicillin allergy was first recorded, and the date the penicillin allergy record was updated. Data were filtered to include the following provider types: resident physician, nurse practitioner, physician assistant, and attending physician.

### Analysis

We used the Revised Standards for Quality Improvement Reporting Excellence (SQUIRE) 2.0 guidelines to report our findings [16]. The most commonly prescribed NBLs were included for analysis. A list of β-lactam and non-β-lactam antibiotics included are in Table 1. Several endpoints were collected for analysis: percent of β-lactams administered before and after intervention (% of total antibiotic administrations for antibiotics included in the analysis (Table 1), nonspecific PCN reaction types (defined as “Other” or “Unknown” documented for penicillin allergy reaction type), number of BPA fires, and number of ‘anaphylaxis order sets’ ordered after BPA fire.

**Table 1:** Antibiotics included in analysis

β-lactams	Non-β-lactams
Amoxicillin	Amikacin
Ampicillin	Azithromycin
Cefaclor	Aztreonam
Cefazolin	Ciprofloxacin
Cefdinir	Clarithromycin
Cefepime	Clindamycin
Cefoxitin	Colistin
Cefpodoxime	Doxycycline
Ceftaroline	Erythromycin
Ceftazidime	Fosfomycin
Ceftolozane	Gatifloxacin
Ceftriaxone	Gentamicin
Cefuroxime	Levofloxacin
Cephalexin	Linezolid
Ertapenem	Metronidazole
Imipenem	Minocycline
Meropenem	Moxifloxacin
Nafcillin	Norfloxacin
Oxacillin	Ofloxacin
Penicillin	Sulfamethoxazole
Piperacillin	Tetracycline
	Tobramycin
	Vancomycin

Antibiotic prescribing rates over time were categorized based on whether the antibiotic was a β-lactam or a non-β-lactam, and reported as percentage of all antibiotic doses included in the analysis. All categorical outcome analyses comparing pre-intervention data to post-intervention data were performed with Chi-square tests. All analyses were done using R version 4.0.3 [17] and R studio version 1.3.1093 [18].

The project was deemed a quality project by the Quality Improvement Committee in the Department of Medicine, and thus Institutional Review Board review was not required. The project received funding from the Society of Hospital Medicine Student Hospitalist Scholar Grant Program.

## Results

There were 4115 antibiotic administrations for 969 patients in the 18 month-pre-intervention period and 1310 antibiotic administrations for 355 patients in the 9-month period included in our impact analysis. Univariate comparison of summary characteristics (Table 2) showed that patients did not differ significantly by age or sex.

**Table 2:** Summary statistics comparing pre-intervention antibiotic administration data with post-intervention antibiotic administration data for patients with penicillin allergies

	Pre-intervention	Post-intervention	*p* value
Antibiotic doses administered	4115	1310	N/A
Patients who received antibiotics	969	355	N/A
Age [mean (SD)]	64.79 (17.62)	63.80 (17.50)	0.079
Sex (%)			0.667
Female	2727 (66.3)	859 (65.6)	
Male	1388 (33.7)	451 (34.4)	
Allergy severity (%)			0.026
High	974 (41.5)	347 (43.3)	
Medium	267 (11.4)	64 (8.0)	
Low	1107 (47.1)	390 (48.7)	
Reaction type (%)			0.084
Systemic	276 (53.9)	103 (61.3)	
Topical	79 (15.4)	26 (15.5)	
Side effect	74 (14.5)	26 (15.5)	
Intolerance	47 (9.2)	9 (5.4)	
Not verified	36 (7.0)	4 (2.4)	

Due to many patients receiving multiple antibiotic orders, the table displays both total administered doses as well as the number of unique patients who received antibiotics

**Fig. 2:**
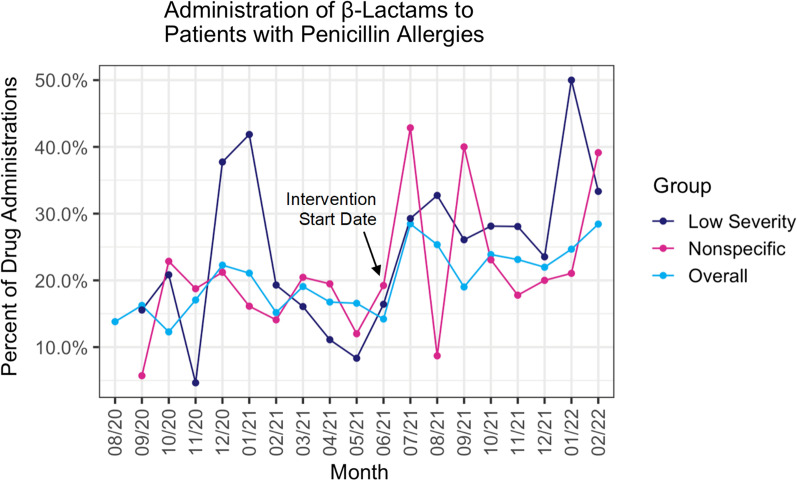
This figure visualizes a 19-month period of β-Lactam administration to various patient subgroups with documented penicillin allergies. Comparing data from before the intervention to after the intervention, there was a statistically significant increase in the percentage of β-Lactam administration for three separate analysis subgroups: patients with a low severity allergy reaction to penicillin (20% to 28%, *p* = 0.0007), patients with a nonspecific penicillin allergy reaction type (19% to 25%, *p* = 0.032), and overall for all patients with a documented penicillin allergy (19% to 23%, *p* = 0.006)

There was an increase in the percentage of orders for β-lactam antibiotics for patients with a documented penicillin allergy after the intervention, from 19 to 23% (*p* = 0.006). Subgroup analyses found an increase in β-lactam administration for patients with a documented nonspecific penicillin allergy reaction type (from 19 to 25% after the intervention, *p* = 0.032) and for patients with a low severity penicillin allergy (from 20 to 28%, *p* = 0.0007). These three significant increases in β-lactam administration are visualized in Fig. 2. There was also a statistically significant decrease in nonspecific PCN allergy reactions listed, from 23% in the pre-intervention period to 20% post-intervention (*p* = 0.012).

Overall, the BPA fired 11 times between June 1, 2021 and February 28, 2022. There were no changes from Aztreonam to a β-lactam antibiotic in the immediate period after the BPA fired. There were no orders of the anaphylaxis order set.

## Discussion

Our multidisciplinary student-led, multipronged intervention focusing on targeted clinician education modules, including peer-to-peer information sharing and in-person small group discussions, and CDS strategies delivered through the EMR increased prescribing of β-lactam antibiotics and decreased documentation of nonspecific PCN allergies.

Wanat and colleagues found that health care prescribers were hesitant to change patient allergy records based on assessment alone and were unsure of testing and referral criteria for patients with suspected penicillin allergy [19]. Other studies have found that prescribers were reluctant to change medical records even when they suspected patients did not have a true allergy due to missing information in patient’s medical records such as reaction details [20, 21]. Our results show that an educational intervention addressing prescriber concerns and a paired EMR intervention decreased the use of NBLs and decreased the rate of nonspecific PCN allergy reaction documentation in the EMR. Though we are unable to discern whether the improved outcomes were primarily due to the educational component or the EMR enhancements, the timely provision of relevant information, inclusion of residents to conduct peer-to-peer communications, and ease of access to enduring resources may have contributed to the intervention’s success. Our didactic sessions focused on providing best practices for caring for patients with a documented low-risk penicillin allergy and the importance of taking a robust allergy history and incorporating findings into the EMR. In addition, the document reviewed during didactic sessions was embedded in the allergy history record in the EMR for ease of reference when prescribing for patients with a penicillin allergy. This “just-in-time” education provided as part of the EMR intervention may have contributed to improved performance compared to conventional educational interventions which have been previously described in the literature.

The BPA for patients prescribed aztreonam did not lead prescribers to select a β-lactam instead of an NBL for patients who had tolerated a β-lactam in the past. However, we are unable to draw conclusions on the impact due to the limited number of instances the BPA fired.

Strengths of our project include the simple intervention design and implementation of the intervention by nursing and medical students. The clarity of the educational sessions and CDS elements allowed our team to leverage team members who were relatively early in their training. The particular EMR intervention chosen emphasized “just-in-time” education for prescribers, and as such decreased the need for more intensive ongoing in-person educational sessions. The likelihood of sustainability of the intervention is high because of stronger reliance on the CDS compared to the in-person educational sessions, which were offered for only two months during a period of transition of staff and trainees (i.e. over the summer months). The CDS can help reinforce the learnings from the in-person educational sessions. In addition, annual educational interventions focused on new trainees can be considered to maintain a culture of antibiotic stewardship.

Our team of students was set up for success by having strong faculty support through the Student High Value Care Curriculum, with one faculty mentor and one resident mentor. This structure allows students to easily access feedback and strategic direction from the populations they are targeting for the intervention. In addition, our resident mentor helped circulate our project with their colleagues, further reinforcing our message. In addition, having a nursing student on our team enabled excellent access to nursing leads and staff to facilitate communication about the project and to schedule trainings. Supportive faculty, residents, and clinicians are key to success with student-led interventions.

The COVID-19 pandemic could have impacted our results. We attempted to address this by gathering baseline data that included pre-pandemic prescribing numbers. Our intervention results do include the winter 2021 COVID-19 surge. We tried to reduce the impact this would have on our results by not including ICU patients in our patient population. With patients that are critically ill, providers might be less willing to investigate if a Penicillin allergy is low-risk. Our patient population is from the medicine service only. COVID-19 positive patients were admitted to the general medicine service throughout our intervention in isolation rooms. Future studies post-pandemic can clarify the impact, if any, the winter 2021 COVID-19 surge had on our data.

Our study has several limitations. First, the non-randomized design does not allow us to draw definitive conclusions on the efficacy of our intervention. However, the similar patient populations in the pre- and post-intervention periods without other clear confounders suggest efficacy of the intervention. Second, as the intervention was limited to the hospital medicine service, it remains to be seen whether this intervention would be equally effective on non-medical services. Lastly, due to EMR limitations, we were unable to track activity related to the embedded link on the allergy page that includes antibiotic prescribing best practices. Future studies should examine the efficacy of a similar intervention in other environments, such as emergency medicine and surgical settings.

## Conclusion

Our intervention combining clinician education and CDS through the EMR decreased the use of non-specific PCN reaction documentation and increased use of β-lactam antibiotics for patients with low-risk PCN allergies. Reducing prescribing of unnecessary NBLs for patients with low-risk PCN allergies can help the healthcare community achieve its goal of improved antibiotic stewardship and reduce poor patient outcomes.

## Supplementary Information

**Additional file 1**. The figure on this page displays best practices for antibiotic prescribing, represented as an algorithm developed by the antibiotic stewardship committee.

## Data Availability

The datasets used and/or analysed during the current study are available from the corresponding author on reasonable request.

## Abbreviations

BPA: Best practice advisory
CDS: Clinical decision support
EMR: Electronic medical record
MRSA: Methicillin-resistant Staphylococcus aureus
VRE: Vancomycin-resistant enterococcus

## References

[1] Browne AJ, Chipeta MG, Haines-Woodhouse G (2021). Global antibiotic consumption and usage in humans, 2000–18: a spatial modelling study. Lancet Planet Health.

[2] Germovsek E, Barker CI, Sharland M (2017). What do I need to know about aminoglycoside antibiotics?. Arch Dis Childhood-Educ Pract.

[3] Lobanovska M, Pilla G (2017). Focus: drug development: Penicillin’s discovery and antibiotic resistance: lessons for the future?. Yale J Biol Med.

[4] Castells M, Khan DA, Phillips EJ (2019). Penicillin allergy. N Engl J Med.

[5] Staicu ML, Soni D, Conn KM, Ramsey A (2017). A survey of inpatient practitioner knowledge of penicillin allergy at 2 community teaching hospitals. Ann Allergy Asthma Immunol.

[6] American Academy of Allergy A, American College of Allergy A, Joint Council of Allergy A, Parameters JTFoP (2010). Drug allergy: an updated practice parameter. Ann Allerg Asthma Immunol Off Publ Am Coll Allerg Asthma Immunol..

[7] Picard M, Bégin P, Bouchard H (2013). Treatment of patients with a history of penicillin allergy in a large tertiary-care academic hospital. J Allerg Clin Immunol.

[8] Macy E, Contreras R (2014). Health care use and serious infection prevalence associated with penicillin “allergy” in hospitalized patients: a cohort study. J Allerg Clin Immunol.

[9] Blumenthal KG (2018). Risk of meticillin resistant Staphylococcus aureus and Clostridium difficile in patients with a documented penicillin allergy: population based matched cohort study. BMJ.

[10] Joseph Mattingly T, Fulton A, Lumish RA, Williams AMC, Yoon SJ, Yuen M, Heil EL (2018). The cost of self-reported penicillin allergy: a systematic review. J Allerg Clin Immunol Pract.

[11] Fuller RN, Baker MG, Desai MB (2021). Overcoming challenges to removing inappropriate penicillin allergy labels: a quality improvement report. Antimicrob Steward Healthc Epidemiol.

[12] Khan DA. Proactive management of penicillin and other antibiotic allergies. 2020:10.2500/aap.2020.41.190024 32122444

[13] North L (2021). Strategies to identify and prevent penicillin allergy mislabeling and appropriately de-label patients. J Family Pract.

[14] Brown CA (2018). Reducing outpatient antibiotic prescribing for acute respiratory infections: a quasi-experimental study. J Doct Nurs Pract.

[15] Frost HM, Andersen LM, Fleming-Dutra KE, Norlin C, Czaja CA (2020). Sustaining outpatient antimicrobial stewardship: do we need to think further outside the box?. Infect Control Hosp Epidemiol.

[16] Ogrinc G, Davies L, Goodman D, Batalden P, Davidoff F, Stevens D (2016). SQUIRE 2.0 (standards for quality improvement reporting excellence): revised publication guidelines from a detailed consensus process. J Nurs Care Qual.

[17] Team RC. R Core Team R: a language and environment for statistical computing. Foundation for Statistical Computing. 2020;

[18] Team R. RStudio Version 3.60: integrated development for R. RStudio Inc. 2020;

[19] Wanat M, Anthierens S, Butler CC, Savic L, Savic S, Pavitt SH, Sandoe JAT, Tonkin-Crine S (2019). Patient and primary care physician perceptions of penicillin allergy testing and subsequent use of penicillin-containing antibiotics: a qualitative study. J Allerg Clin Immunol Pract.

[20] Ouazana A, François M, Pung R, Dona M, Jami A (2015). Conduites des médecins face aux allergies médicamenteuses. Attitudes comparées entre médecins généralistes et allergologues. Étude qualitative. Rev Fr Allergol.

[21] Wanat M, Anthierens S, Butler CC (2021). Management of penicillin allergy in primary care: a qualitative study with patients and primary care physicians. BMC Fam Pract.

