# Supplementary material for: An interdisciplinary student-led multifaceted intervention addressing overuse of broad-spectrum antibiotics for patients with penicillin allergies
Source: Antimicrob Resist Infect Control. 2023 Apr 15;12:34. doi: 10.1186/s13756-023-01232-0 (PMC10105531; doi:10.1186/s13756-023-01232-0)
Supplement: Supplementary file 1 — Additional file 1. The figure on this page displays best practices for antibiotic prescribing, represented as an algorithm developed by the antibiotic stewardship committee. [file 13756_2023_1232_MOESM1_ESM.pdf]

# Antibiotic Prescribing Guidelines for Documented PCN Allergy

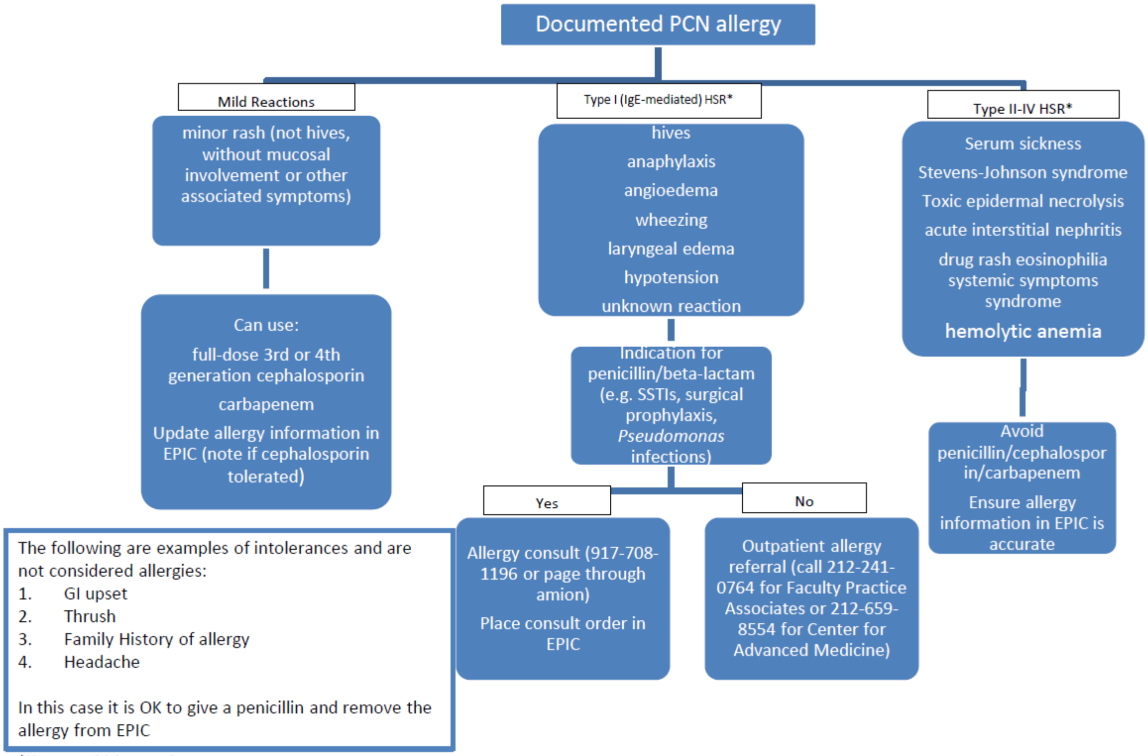


*Hypersensitivity reaction

**See reverse side for details on taking an allergy history

***This is meant to serve as a general patient guideline, not a substitute for clinical judgment. If you are ever unsure or uncomfortable, consult allergy/immunology or infectious diseases.
